# Light Chain Separated from the Rest of the Type A Botulinum Neurotoxin Molecule Is the Most Catalytically Active Form

**DOI:** 10.1371/journal.pone.0012872

**Published:** 2010-09-22

**Authors:** Nizamettin Gul, Leonard A. Smith, S. Ashraf Ahmed

**Affiliations:** 1 Integrated Toxicology Division, Department of Cell Biology and Biochemistry, United States Army Medical Research Institute of Infectious Diseases, Fort Detrick, Maryland, United States of America; 2 Medical Countermeasures Technology, United States Army Medical Research and Materiel Command, Fort Detrick, Maryland, United States of America; University Paris Diderot-Paris 7, France

## Abstract

Botulinum neurotoxins (BoNT) are the most potent of all toxins. The 50 kDa N-terminal endopeptidase catalytic light chain (LC) of BoNT is located next to its central, putative translocation domain. After binding to the peripheral neurons, the central domain of BoNT helps the LC translocate into cytosol where its proteolytic action on SNARE (soluble NSF attachment protein receptor) proteins blocks exocytosis of acetyl choline leading to muscle paralysis and eventual death. The translocation domain also contains 105 Å -long stretch of ∼100 residues, known as “belt,” that crosses over and wraps around the LC to shield the active site from solvent. It is not known if the LC gets dissociated from the rest of the molecule in the cytosol before catalysis. To investigate the structural identity of the protease, we prepared four variants of type A BoNT (BoNT/A) LC, and compared their catalytic parameters with those of BoNT/A whole toxin. The four variants were LC + translocation domain, a trypsin-nicked LC + translocation domain, LC + belt, and a free LC. Our results showed that *K_m_* for a 17-residue SNAP-25 (synaptosomal associated protein of 25 kDa) peptide for these constructs was not very different, but the turnover number (*k*_cat_) for the free LC was 6-100-fold higher than those of its four variants. Moreover, none of the four variants of the LC was prone to autocatalysis. Our results clearly demonstrated that *in vitro*, the LC minus the rest of the molecule is the most catalytically active form. The results may have implication as to the identity of the active, toxic moiety of BoNT/A *in vivo*.

## Introduction

Seven immunologically distinct neurotoxins, produced by strains of *Clostridium botulinum*, are most potent of all toxins (for a review see references [1], [2]. Botulinum neurotoxins (BoNTs) are composed of three major structural domains of approximately equal size of 50-kDa each. They are an N-terminal Zn-endopeptidase catalytic domain – called light chain (LC), a central translocation domain (Hn), and a C-terminal receptor binding domain (Hc). The latter two in a single polypeptide is known as heavy chain (HC). A segment of the central translocation domain, called a belt, wraps around the LC so that the access to its active-site is occluded from solvent [3], [4]. Intoxication of cells by BoNT is believed to be mediated sequentially by receptor binding by Hc, endocytotic pore formation by Hn, translocation of the catalytic LC domain into the cytosol, and proteolysis of SNARE proteins resulting in disruption of acetylcholine release in exocytosis leading ultimately to muscle paralysis and death [1], [2]. In our continuing efforts to understand the structural contribution on the enzyme's catalytic mechanism, we have studied its autocatalytic reactions [5], [6], [7], [8], active site residues [5], 3-D structures [9], [10], [11], and ability of expressed LC in inhibiting exocytosis in sea urchin eggs [12]. There is however, no experimental evidence available in the literature if the LC catalytic domain enters the cytosol and functions alone, dissociated from the rest of the molecule. In this paper, we address this question indirectly by analyzing the kinetics of proteolysis reaction catalyzed by the LC alone, LC containing a belt that wraps around it, LC plus Hn domain containing the belt, a trypsin- nicked LC plus Hn', and the LC within the whole BoNT of serotype A.

## Materials and Methods

### BoNT/A LC, chemicals, buffers and reagents

**Figure 1:**
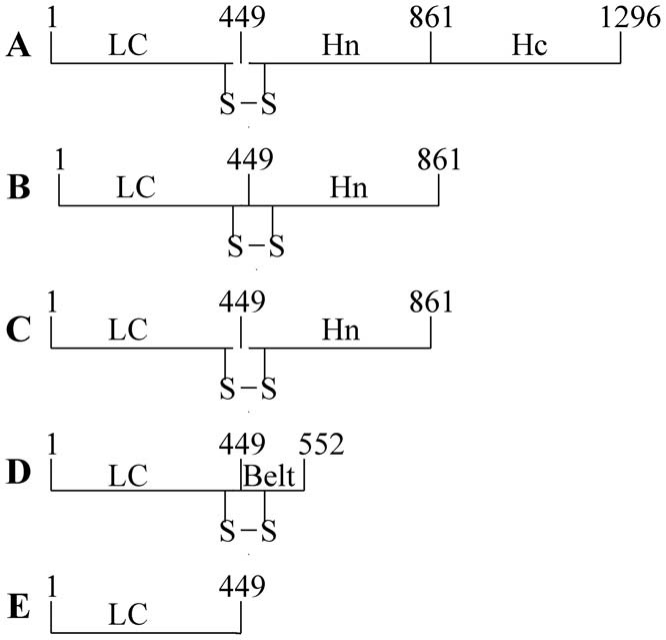
Schematic presentation of LC as it occurs in BoNT/A (residues M1-L1296) (A), LC+Hn (residues (M1-Q861) (B), trypsin-cleaved LC+Hn' (residues M1-Q861) (C), LC+Belt residues M1-F552) (D), and LC alone (residues M1-K449) (E). The numbers represent the sequence stretch of each construct.

The 449-residue recombinant BoNT/A LC with an extra valine residue at position 2 [13] was expressed and purified as described [8]. The homogeneous preparation was stored at −20°C in buffer P (50 mM Na-phosphate pH 6.5) containing 150–250 mM NaCl and 2 mM EDTA. The LC constructs LC+Hn and LC+Belt were purified to homogeneity as described [14]. Nicking of the hinge between LC and Hn domains were achieved by limited proteolysis by TPCK-trypsin as described [14]. Figure 1 shows the schematic representation of the five versions of the LC used in this study.

Proteins were desalted by passing through PD-10 gel filtration columns and were collected in appropriate buffer before each experiment and assays. Buffer P (50 mM Na-phosphate, pH 6.5) was used throughout except in the enzyme assays where 50 mM Na-HEPES pH 7.4 was employed. Full-length BoNT/A was from Metabiologics, Madison, WI. Zinc chloride was obtained from Sigma. Rabbit polyclonal antibodies against a 16-residue N-terminal sequence (PFVNKQFNYKDPVNGV) of BoNT/A LC were produced and affinity-purified by Research Genetics (Huntsville, AL). Affinity-purified, peroxidase-coupled, goat anti-rabbit and anti-mouse IgG (H+L) and ABTS substrate were from Kirkegaard Perry Laboratories (Gaithersburg, MD).

### Enzymatic activity assays

**Figure 2:**
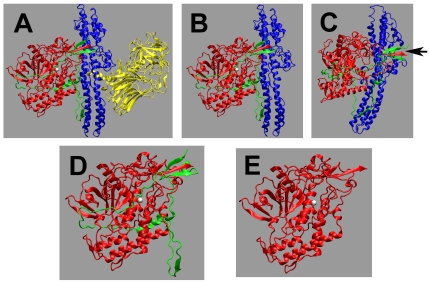
Ribbon diagram of the five versions of the BoNT/A LC used in this study. These diagrams are based on the original 3-dimensional structure of BoNT/A dichain determined by Stevens et al [3]. The constructs used in this study and represented by the structures A, B, and D however are single polypeptide chains. A, Structure of BoNT/A whole toxin. The three major structural domains, the n-terminal LC (red), the central translocation Hn (blue), and the C-terminal binding Hc (yellow) are shown. A stretch of ∼115 residues belonging to the translocation domain Hn that wraps around the LC and known as belt, is shown in green. B, LC+Hn; C, LC+Hn that was nicked by trypsin. Here Figure B is slightly rotated to visualize the tryptic cleavge site indicated by an arrow; D, LC+Belt, and E, LC. Although LC and Hn are shown separated in C to distinguish it from B, these domains are in fact still connected by a disulfide bond (see Figure 1) in addition to other ionic and hydrophobic interactions. Figures B–E were generated by simple truncations from the C-terminus of A.

The enzymatic assay was based on HPLC separation and measurement of the cleaved products from a 17-residue C-terminal peptide corresponding to residue #187–203 of SNAP-25 [13], [15]. A master reaction mixture lacking the LC catalysts was made and aliquots were stored at −20°C. At the time of assay, an aliquot of the master mix was thawed and 25 µl was added to 5 µl of the LC (see above) to initiate the enzymatic reaction. Components and final concentration in this 30-µl reaction mixture were 0.9 mM substrate peptide, 0.25 mM ZnCl_2_, and 0.16–0.55 µM LC, and 50 mM Na-HEPES, pH 7.4. ZnCl_2_ was included because it stimulated the activity of the LcA preparation [5], [13]. After 3–5 min incubation at 37°C, the reaction was stopped by acidifying with 90 µl of 0.7% trifluoroacetic acid (TFA), and 100 µl of this mixture was analyzed by HPLC as described [13]. For *K_m_* and *k_cat_* determinations (Figure 2C), the reaction mixtures were incubated at 23°C.

### Autocatalysis experiments

Before each experiment, aliquots of the LC were thawed to room temperature and immediately passed through a PD-10 gel-filtration column equilibrated with Buffer P. The protein was collected in Buffer P and stored on ice. The LC was mixed with predetermined concentrations of ZnCl_2_ and 50-µl aliquots were distributed in screw-capped Eppendorf tubes. The final concentration of the protein was 0.2 mg/ml in these incubation mixtures. The tubes were incubated at 22°C. At various time intervals 25 µl of 2X SDS-load buffer was added to a 50-µl aliquot for SDS-PAGE analysis.

### SDS-PAGE and Western Blot

SDS-PAGE was carried out under reducing and non-reducing conditions [16] on 1 mm thick 10% tricine-gels (Novex) as described [17]. Samples were boiled for 5 min in 0.4% SDS, 12% glycerol and 450 mM tris-HCl (pH 8.45). Reducing condition was maintained by adding 5% β-mercaptoethanol to the SDS-load buffer. The running buffer contained 0.1% SDS in 0.1 M tris-0.1 M tricine, pH 8.3. The gels were stained with Coomassie Brilliant Blue. Protein bands were scanned in a BioRad GS-710 Densitometer gel scanner with Quantity One software and the relative amount of proteins in stained bands in each lane were measured. Identity of LC and its N-terminal fragments were confirmed by Western blots on nitrocellulose membranes that were prepared by using a primary polyclonal antibody against a 16-residue N-terminal sequence of BoNT/A LC and a peroxidase-coupled, goat anti-rabbit IgG (H+L) as the secondary antibody [13].

### UV-visible absorption, circular dichroism, and fluorescence measurements

To determine protein concentration and to assess purity, UV-visible absorption spectra were recorded at 22°C with a Hewlett-Packard 8452 diode array spectrophotometer. LC concentration was determined using *A* ^0.1%^ (1 cm light path) value of 1.0 at 278 nm [8] or by BCA assay (Pierce) with bovine serum albumin as standard.

Circular dichroism spectra of 0.2 mg/ml of each protein in 50 mM Na-phosphate, pH 6.5, were recorded at 20°C, with a Jasco 718 spectropolarimeter with quartz cuvettes of 1 mm path length. An average of five scans was recorded to increase signal-to-noise ratio at a scan speed of20 nm/min with a response time of 8 sec. In all measurements, a buffer blank was recorded separately and subtracted from sample recordings. Molecular and mean residue weights used, respectively, were LcA 51449.6 and 114.587, LcA+Belt 63411.8 and 114.876, LcA+Hn 98682.1 and 114.613, and LcA+Hn' 98700.1 and 114.634. Secondary structural contents were calculated by SELCON supplied in the Softsec program (Softwood, CO.).

Tryptophan fluorescence emission spectra were recorded at 20°C (10°C for Zn-autocatalyzed LC) in a PTI QuantaMaster Spectrofluorimeter, Model RTC 2000 equipped with a Peltier-controlled thermostat and Felix software package. Emission and excitation slit widths were set at 1 nm and excitation wavelength at 295 nm. Each spectrum was an average of five scans.

## Results

### Structural characterization of the LC variants

Five variants of LcA employed in this study are shown in Figure 2. Four of them, namely LC+Hn (Figure 2B), LC+Hn' (Figure 2C), LC+Belt (Figure 2D), and LC (Figure 2E) were constructed from synthetic genes [14]. The structures in Figure 2B and 2C should be essentially identical having an inter-domain disulfide bond, except that the latter underwent a limited proteolysis at a hinge between the two domains by trypsin.

**Figure 3:**
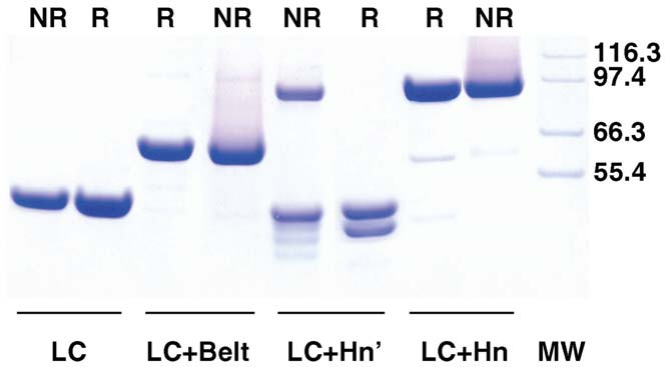
Reducing (R) and non reducing (NR) SDS-PAGE of various forms of the LC used. Samples (0.15 mg/ml) were heated at >95°C for 5 min without (NR) and with (R) 5% β-mercaptoethanol in the SDS-load buffer before electrophoresis. Molecular mass in kDa of the marker proteins in the right lane are shown at right.

Purity and linear structures of the constructed LC variants were probed by SDS-PAGE under reducing and non-reducing conditions (Figure 3). Because LC does not contain a disulfide bond, it migrates as a single band corresponding to a molecular mass of ∼51 kDa under both reducing and non-reducing conditions. LC+Belt, and LC+Hn, on the other hand contain inter-chain disulfide bonds. However, because they each represent single polypeptide chains, their electrophoretic mobility was not affected by reducing condition except that a trailing smudge preceded the major stained bands of approximately 63 kD, and 96 kD, respectively. Faint, stained bands accompanying these two preparations probably represent contaminants, not derived from the BoNT protein, because they were not recognized by two unrelated polyclonal antibodies [14]. They may also represent an insignificant population of the proteins not recognized by the antibody. Electrophoresis under non-reducing condition of trypsin-treated LC+Hn showed two major bands; one of ∼96 kDa expected for LC+Hn, and the other of ∼50 kDa, and three smaller faint bands. Treatment of this construct by β-mercaptoethanol, however, completely reduced it into two bands corresponding to those of the LC and the Hn. In addition to K449-A450 bond, there are four additional tryptic cleavage sites within 10 residues on either side of this bond in the hinge region between LC and Hn [18]. We did not identify the exact location of the tryptic cleavage, but migration of the major band in the LC+Hn' sample under reducing condition along the LC band (Figure 3, and [14]) suggest that the major tryptic cleavage was at K449-A450 bond.

**Figure 4:**
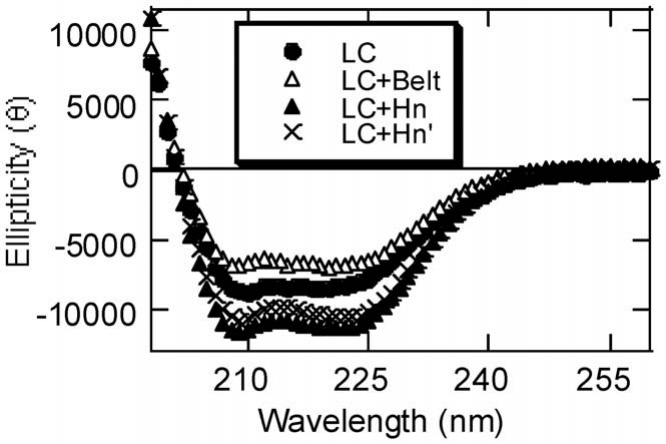
Far-UV CD spectra of LC variants at 20°C. Average of five spectra were collected for 0.2 mg/ml of each of LC (closed circle), LC+Belt (open triangle), LC+Hn (closed triangle), and LC+Hn' (crossed hatch, X) in 50 mM sodium-phosphate, pH 6.5.

**Figure 5:**
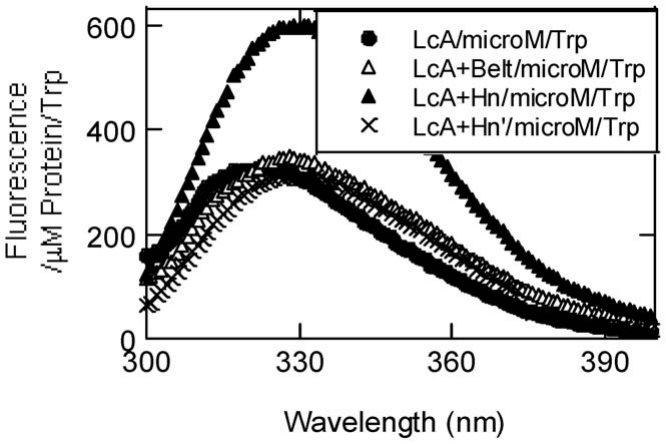
Tryptophan fluorescence spectra of the LC variants at 20°C. Fluorescence emission of each protein collected at 0.20–0.33 mg/ml in 50 mM sodium phosphate, pH 6.5, was adjusted for 1 µM protein/tryptophan. LC (closed circle), LC+Belt (open triangle), LC+Hn (closed triangle), and LC+Hn' (crossed X).

Our SDS-PAGE results under reducing and non-reducing conditions supported the expected linear structures of the LC variants. LC and Hn domains of BoNT proteins have predominantly helical secondary structures [18]. To determine the secondary structural integrity of our four LC variants, we collected their far-UV circular dichroism spectra (Figure 4). The spectra with negative ellipticity peaks at 222 nm and 208 nm showed that the general pattern of all the variants represented helical proteins [19], and their calculated α-helical content (Table 1) was not significantly different than that observed in the crystal structure of BoNT/A [3]. Somewhat lower α-helix content for LC+Belt may suggest an altered structure of the belt region in the absence of the rest of the protein. The hinge between HC and Hn is mostly unstructured in the dichain form of BoNT/A [18]. Therefore its cleavage by trypsin was not expected to change the secondary structures. Yet the calculated α-helical content of HC+Hn' is significantly lower than that of HC+Hn. At present, we do not have an explanation of this unexpected behavior. Nonetheless, we also found significantly high tryptophan fluorescence of LC+Hn compared to that of LC+Hn' (Figure 5).

**Table 1:** Helical content of LC variants compared with values derived from three-dimensional (3-D) and infrared (IR) structures.

LC Variant	CD (This work)	3-D [14]	IR [38]
LC	0.27	0.28	
LC+Belt	0.21	0.28	
LC+Hn	0.36	(0.38)	
LC+Hn'	0.26	0.38	
BoNT/A	n.d.	0.28	0.31

Tertiary structural integrity of the constructs was probed by their intrinsic tryptophan fluorescence spectra (Figure 5). Tryptophan contents per polypeptide of LC, LC+Belt, LC+Hn and LC+Hn' were 2, 3, 6, and 6, respectively. Fluorescence emission results therefore were expressed as fluorescence/µM polypeptide/tryptophan. Free tryptophan has a fluorescence emission maximum at 354 nm (not shown). This maximum shifted to blue for all of the LC variants depending on the location, exposure, direction and distance of the tryptophan residues from polar groups including water [20]. The most notable feature of tryptophan fluorescence was that a single nick at the hinge between LC and Hn domains reduced the fluorescence intensity dramatically. Such change in intensity may indicate perturbation in the inter domain arrangement of the protein. In the absence of similar data with trypsin-nicked LC+Hn' in the literature, we could not make any rational conclusion on the tryptophan fluorescence data.

### Catalytic Activity of the LC variants

**Figure 6:**
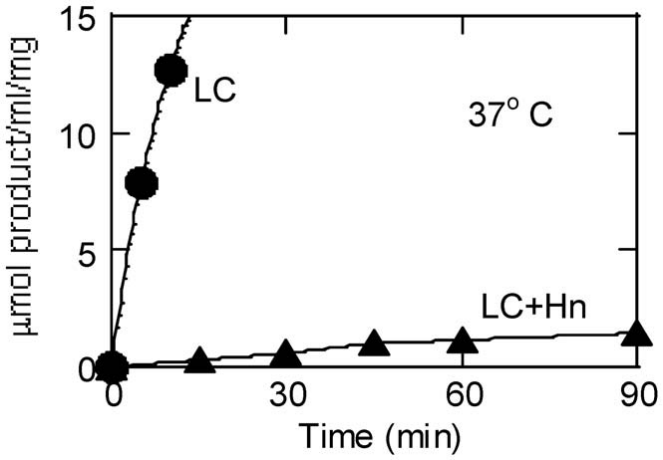
Time course of reactions catalyzed by LC and LC+Hn. LC (0.4 µg, closed circle) or LC+Hn (1.8 µg, closed triangle) and 1 mM peptide substrate in a 30 µl of reaction mixture containing ZnCl_2_ and DTT was incubated at 37°C. At the indicated times, products in two vials was analyzed by HPLC as described in the EXPERIMENTAL PROCEDURES.

We determined the catalytic activity of the LC variants on a 17-mer peptide substrate in three different conditions. Because the BoNT LC is a Zn-containing and Zn-dependent endopeptidase, enhancement of its catalytic activity was observed in some preparations by adding exogenous zinc (0.25 mM ZnCl_2_) depending on its zinc content. LC+Belt and LC+Hn variants contain a disulfide bond between the LC moiety and the rest of the molecules as in all full-length BoNTs. Including up to 5 mM dithiothreitol (DTT) was reported necessary for significantly increasing catalytic activity of full-length BoNT/A [21]. Thus, the activity of the latter two LC variants was also determined in the presence of 0.25 mM ZnCl_2_ plus 5 mM DTT. The highest values in activity are shown in Table 2. Figure 6 compares the amount of products formed from the peptide substrate by LC+Hn with that by LC in the presence of ZnCl_2_ and DTT as a function of time. Including ZnCl_2_ and DTT in this experiment was to ensure availability of Zn, should it dissociate from Lc+Hn during the prolonged incubation. On a weight basis, the LC+Hn was a much weaker catalyst. Fairly linear progress curve with LC+Hn also indicated that this construct was quite stable over the course of prolonged incubation at 37°C. Curvature of LC reaction was due to sub-*K_m_* (Table 2) concentration of substrate used. Although a trypsin-nicked LC+Belt was not prepared due to its poor yield [14], we do not anticipate activity results much different than that obtained with trypsin-nicked LC+Hn.

**Table 2:** Specific activity and kinetic constants of various light chain constructs.

LC variant	Sp. Act. (µmol/min/mg)	Rel. Sp. Act.	*K_m_* (mM)	*k_cat_* (Sec^−1^)	Rel. *k_cat_*	*k_cat_/K_m_*
LC	2.15±0.01	100	3.36	9.00	100	2.68
LC+Belt	0.27±0.02	15.0	1.30	0.31	3.4	0.24
LC+Hn'	0.09±0.00^1^	8.2	n.d.	n.d.	-	-
LC+Hn	0.02±0.00	1.6	0.8	0.09	1.0	0.11
BoNT/A	0.09±0.01	12	4.29	1.42	15.8	0.33

**Figure 7:**
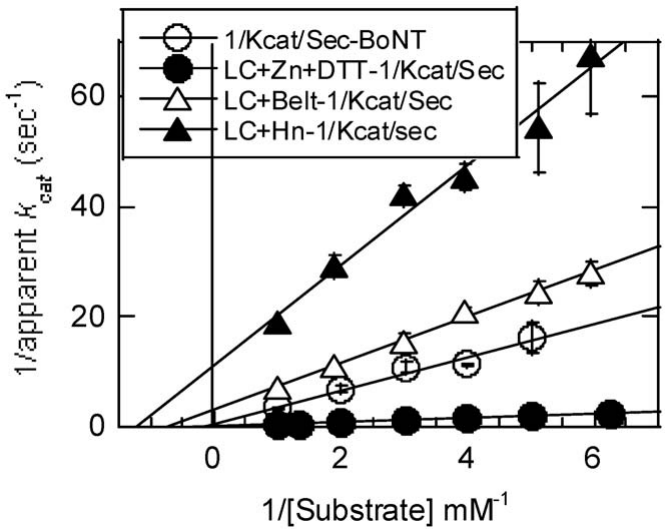
Lineweaver-Burke plots of reaction velocity versus substrate concentration of reactions catalyzed by three LC variants. The bars accompanying the data points are standard deviations of three to five assays. LC (closed circle), LC+Belt (open triangle), LC+Hn (closed triangle), and BoNT/A (open circle).

Specific activity (Sp. Act.) was determined at a fixed substrate concentration of 0.9 mM in triplicate. *K_m_* and *k_cat_* values were calculated from double reciprocal plots shown in Figure 7.

Table 2 lists the specific activities of the LC variants, along with that of whole BoNT/A, at a fixed concentration of substrate. A highest activity of 2.15 µmol/min/mg was obtained with the LC. This value is more than eightfold higher than the activity of full-length BoNT/A, and is at least 10 times higher than BoNT/A activity reported in the literature [15], [21]. Specific activity of the LC+Hn variant was less than 25% of that obtained with its trypsin-nicked counterpart. The result indicates the necessity of nicking between the LC and the rest of the molecule to express its optimum catalytic activity.

### Kinetic constants

To compare the catalytic efficiencies of the LC variants, we determined their substrate concentration at half maximal velocity, *K_m_*, and catalytic rate constant, *k_cat_*, from double reciprocal, Lineweaver-Burke plots (Figure 7). As shown in Table 2, *K_m_* for the smallest LC and the largest, full-length BoNT/A were high and similar, but that for the Lc+Belt and LC+Hn were small. Lower *K_m_* of the latter two constructs, however, also resulted in a significantly lower catalytic turnover (*k_cat_*) making them poorest of the catalysts. LC+Belt and LC+Hn were poorest of the five catalysts. In the absence of most of the HC and of Hc in these two constructs, we could not rule out the possibility that the active site or its opening was not adversely affected by the truncations. Because of the difficulties in obtaining sufficient quantities of trypsin-nicked, LC+Hn, we could not determine its kinetic constants. However, by comparing its specific activity with that of others, its *k_cat_* was expected to be as high as that of the full-length BoNT/A. In any case, the catalytic efficiency, measured by *k_cat_/K_m_*, of LC alone is 8-24-fold higher than its larger versions.

### Autocatalytic activity of the LC variants

**Figure 8:**
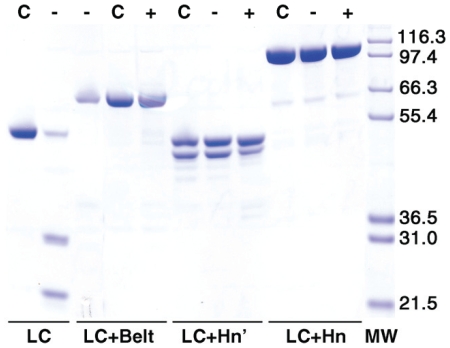
Reducing SDS-PAGE of LC variants before (C), and after 48-h incubation at 22°C in the absence (−) and presence (+) of 5 mM DTT. Samples (0.15 mg/ml) were heated at >95°C for 5 min in the SDS-load buffer containing 5% β-mercaptoethanol before electrophoresis. Molecular mass in kDa of the marker proteins in the right lane are shown at right.

In an earlier study, we demonstrated that the LC undergoes autocatalytic cleavage at various positions depending on solution conditions [5], [6], [8]. Two factors that significantly affect the reactions are low pH and presence of zinc [7]. Figure 8 shows that when the LC was incubated for 48 h in the presence of 0.5 mM ZnCl_2_, it was converted into two major products. On the other hand, none of the other four constructs was prone to autocatalysis either in the presence or in the absence of 5 mM DTT. DTT was added to ensure reduction of the interchain disulfide bond. Even in the reduced, dichain form, LC+Hn did not show any sign of autocatalytic fragmentation.

## Discussion

Botulinum neurotoxin is a three-domain protein having its Zn-protease activity located at the N-terminus. Primarily based on the mechanism of cellular action of diphtheria toxin, it was postulated that after binding the endosome surface with its C-terminal Hc domain, the central translocating Hn domain would make a transmembrane conduit for the N-terminal protease domain to be exposed into the cytosol. Mechanism of Hn-surface binding has been extensively studied by various investigators [22], [23], [24], [25], [26], [27]. The process of translocation has also been addressed by clever experiments by Montal and associates who made several truncated versions of BoNT/A to demonstrate that LC+Hn was sufficient for translocation into the cytosol [28] and used inhibitors to determine the dynamics of translocation [29]. Earlier, they also demonstrated that reduction of the disulfide bond between LC and Hn was essential for the translocation by measuring the catalytic activity of the protease domain [30], [31]. Three-dimensional structure of several serotypes of BoNT has shown a large interaction surface area between the LC and HC domains [3], [4], [10], [32]. Therefore, a simple disulfide reduction would not be expected to dissociate the two chains. Although it has long been known that the disulfide reduction dramatically accelerates the protease activity, the question that remains unanswered is: Is physical dissociation of LC from the rest of the molecule a necessary step in expressing its protease activity within the cytosol?

In this paper, we used five versions of the LC starting with the full-length BoNT/A to the smallest, free LC, and determined their catalytic properties. Reducing and non-reducing SDS-PAGE, absorbance, fluorescence, and CD properties of these LC versions supported their expected gross structures. Therefore, results of the catalytic activity should not be subjected to undue structural influences. Our assays contained DTT as the reducing agent; therefore, the disulfide bond between LC and belt, LC and Hn and LC and HC (in BoNT/A) were reduced in the four larger versions of BoNT/A. From the three-dimensional structure, we know that the belt on the LC covers the active site gorge that will restrict substrate entry or product release. Therefore, lower catalytic efficiency of BoNT/A, LC+Hn, and LC+Belt than the LC alone may most likely be due to shielding of the active site by the belt. A ‘chaperone’ role of the belt for the LC has been proposed based on X-ray structure [33].

A very significant observation is the trypsin-nicked LC+Hn' failed to undergo autocatalytic fragmentation, even though the Hn moiety containing the Belt was not covalently linked to LC (Figure 8). Our results extended the earlier observations by DasGupta [34], [35] that BoNT/A does not undergo autocatalysis with an implication that shielding of the active site by the belt in these constructs prevents this unwanted reaction. Our results prove that complete removal of the belt is necessary for the autocatalytic reaction to occur. Our autocatalytic results thus complemented poor catalytic activity of versions larger than LC.

Activation of the LC protease domain of BoNT toxins by an exogenous protease has long been known [1]. A fourfold increase in specific activity resulted from cleavage of the hinge between LC and Hn of the LC+Hn version. Cleavage of the hinge may partially remove the belt from the active site entry. The fact that the LC alone is 8–24-fold more efficient as a catalyst (Table 2) proves that a physically dissociated LC from the rest of the molecule is the most active catalyst.

In this study we used a 17-mer peptide substrate while use of full-length SNAP-25 would have been the ideal. SNAP-25 lowers the *K_m_* but does not significantly affect the *k_cat_* [5], [7], [36], [37], [38], [39], [40], [41]. Thus, *k_cat_* (4, 11, 50, & 60/sec for LcA or BoNT/A) with the SNAP-25 substrate however is not much different than the 17-mer substrate (5 & 12/sec for LcA & BoNT/A) [5], [7], [36], [37], [38], [39], [40], [41]. The larger SNAP-25 substrate lowers the *K_m_* to 10–50 µM from 3–5 mM for the 17-mer substrate. The only information lacking in this study is the *K_m_* values for the natural substrate, which does not compromise with our obtained *k_cat_* values.

Evolution shapes biomolecules to perform optimally and leads to structural, functional, and catalytic perfection [42], [43], [44] as has been claimed only in case of triosephosphate isomerase [42]. While far from perfection, the LC may have evolved to this dissociated stage to express its protease activity most efficiently. While a structural form displaying the highest catalytic efficiency exists, it is very unlikely that a larger, less active structure would be responsible for what is known as the only ‘physiological’ function of BoNT toxins. Based on these considerations, our results support the idea that a LC dissociated from the Hn domain is the most likely catalyst in the cytosol.
